# Establishment of *Agrobacterium*-Mediated Transient Transformation System in Desert Legume *Eremosparton songoricum* (Litv.) Vass.

**DOI:** 10.3390/ijms252211934

**Published:** 2024-11-06

**Authors:** Xi’an Lao, Pei Jin, Ruirui Yang, Yuqing Liang, Daoyuan Zhang, Youling Zeng, Xiaoshuang Li

**Affiliations:** 1 Xinjiang Key Laboratory of Biological Resources and Genetic Engineering, College of Life Science and Technology, Xinjiang University, Urumqi 830046, China; laoxian010203@163.com; 2 State Key Laboratory of Desert and Oasis Ecology, Key Laboratory of Ecological Safety and Sustainable Development in Arid Lands, Xinjiang Institute of Ecology and Geography, Chinese Academy of Sciences, Urumqi 830011, China; jinpei22@mails.ucas.ac.cn (P.J.); yangruirui19@mails.ucasac.cn (R.Y.); lyuqing007@ms.xjb.ac.cn (Y.L.); zhangdy@ms.xjb.ac.cn (D.Z.); 3 University of Chinese Academy of Sciences, Beijing 100049, China; 4 Xinjiang Key Lab of Conservation and Utilization of Plant Gene Resources, Xinjiang Institute of Ecology and Geography, Chinese Academy of Sciences, Urumqi 830011, China

**Keywords:** *Eremosparton songoricum* (Litv.) Vass., transient transformation, *Agrobacterium*, woody legume, drought tolerance

## Abstract

*Eremosparton songoricum* (Litv.) Vass. is a desert legume exhibiting extreme drought tolerance and the ability to withstand various harsh environments, making it a good candidate for investigating stress tolerance mechanisms and exploring valuable stress-resistant genes. However, the absence of a genetic transformation system for *E. songoricum* poses significant limitations for functionally validating these stress-resistant genes in situ. In this study, we developed an *Agrobacterium*-mediated transient transformation system for *E. songoricum* utilizing the β-glucuronidase (*GUS*) gene as a reporter. We investigated three types of explants (seedlings, assimilated branches and callus) and the effects of different *Agrobacterium* strains, seedling ages, OD_600_ values, acetosyringone (AS) concentrations, sucrose concentrations and infection times on the transformation efficiency. The results reveal that the optimal transformation system was infecting one-month-old regenerating assimilated branches with the *Agrobacterium* strain C58C1. The infection solution comprised 1/2 MS medium with 3% sucrose and 200 μM AS at an OD_600_ of 0.8, infection for 3 h and then followed by 2 days of dark cultivation, which achieving a maximum transformation rate of 97%. The maximum transformation rates of the seedlings and calluses were 57.17% and 39.51%, respectively. Moreover, we successfully utilized the assimilated branch transient transformation system to confirm the role of the previously reported transcription factor EsDREB2B in *E. songoricum*. The overexpression of *EsDREB2B* enhanced drought tolerance by increasing the plant’s reactive oxygen species (ROS) scavenging capacity in situ. This study established the first transient transformation system for a desert legume woody plant, *E. songoricum*. This efficient system can be readily applied to investigate gene functions in *E. songoricum*. It will expedite the exploration of genetic resources and stress tolerance mechanisms in this species, offering valuable insights and serving as a reference for the transformation of other desert plants and woody legumes.

## 1. Introduction

*Eremosparton songoricum* (Litv.) Vass. is a critically endangered leguminous shrub found in Central Asia, with sporadic distributions in the mobile dunes of the Gurbantunggut Desert in Xinjiang, China [1]. Recognized as a typical representative of drought-tolerant species in desert ecosystems [2], *E. songoricum* has developed various morphological adaptations to cope with extreme environmental stresses, such as drought, temperature extremes and UV radiation [3,4]. Specifically, its leaves undergo severe degeneration, transforming into assimilated branches covered with thick, scale-like cuticles that effectively reduce water loss while maintaining efficient photosynthesis. Additionally, this species employs asexual reproduction through horizontal rhizomes [5,6], which optimizes water usage and facilitates the rapid expansion of its reproductive range, thereby contributing to the stability of desert ecosystems by improving soil structure, fixing sand and contributing to material circulation and energy flow. A high-throughput genomics platform for *E. songoricum* has been established, resulting in the acquisition of a high-quality genome (National Gene Bank of China, Project No. CNP0002419) and transcriptome data under drought stress conditions (National Gene Bank of China, Project No. CNP0003150). Analysis of these transcriptomic data has revealed that the AP2/ERF transcription factor family plays a critical role in the drought tolerance of *E. songoricum* [7], with the *EsDREB2B* gene significantly enhancing tolerance to drought, high salinity, cold and heat stress in yeast and transgenic tobacco [8]. These findings lay a solid foundation for further molecular biological research, demonstrating that *E. songoricum* is an excellent resource for gene mining. Furthermore, the successful establishment of regeneration system for *E. songoricum* provides the essential groundwork for subsequent genetic transformation and functional analysis of its resistance genes [9].

The technique of *Agrobacterium*-mediated plant genetic transformation utilizes *Agrobacterium* to transfer and integrate exogenous genes into genomes, producing transgenic plants [10,11]. This method is renowned for its efficiency, simplicity and stability, making it suitable for dicotyledonous plants and some monocotyledonous plants [12]. Following the successful transfer of foreign genes into plants, gene expression can be classified as either transient or stable. Transient gene transformation serves as an effective tool for identifying the function of exogenous genes without affecting plant growth and development. It has been widely applied in plant research and production practice [13]. Meanwhile, stable genetic transformation involves the integration of exogenous genes into the host plant genome, ensuring their expression in subsequent generations [11]. This approach is crucial for plant breeding and genetic engineering. However, developing efficient and reproducible stable transformation techniques for woody plants remains challenging due to limitations such as long regeneration cycles and high genomic heterozygosity. Moreover, with advancements in sequencing technologies and improved genomic assembly, the number of sequenced woody plants now exceeds those with established stable genetic transformation systems [14]. Research has shown that transient genetic transformation has been successfully applied to various woody species, including apricot (*Prunus armeniaca* L.) [15], *Cinnamomum camphora* [16], apple (*Malus domestica*) [17], persimmon (*Diospyros kaki* Thunb) [18], tamarisk (*Tamarix hispida*) [19], birch (*Betula platyphylla*) [19], willow (*Salix matsudana*) [19] and aralia (*Aralia mandshurica*) [19]. *Agrobacterium*-mediated plant genetic transformation significantly shortens breeding cycles compared with traditional breeding methods, especially for woody plants. Effective transient transformation systems and efficient regeneration systems play crucial roles in the rapid screening and validation of gene functions. They allow for a quick assessment of how introduced genes affect plant physiology and development, which is particularly important in woody plants where traditional breeding methods can be slow and less efficient [19].

*E. songoricum* represents a valuable resource for studying stress resistance mechanisms and identifying stress-tolerant genes in desert woody legumes [8]. However, the lack of a genetic transformation system has significantly limited the efficiency of exploring and utilizing stress-resistant genes in *E. songoricum*. We established an *Agrobacterium*-mediated transient transformation system for *E. songoricum* to overcome this limitation. Our research focused on establishing transient genetic transformation in *E. songoricum* by investigating influencing factors on *Agrobacterium*-mediated transformation efficiency, including the strain type, *Agrobacterium* density (OD_600_), infection time and acetosyringone (AS) and sucrose concentrations, using three types of explants: seedlings, assimilating branches and callus. The transformation method using assimilating branches as explants was then employed to validate the function of the *EsDREB2B* gene in response to drought. The *Agrobacterium*-mediated transient transformation method proved to be a rapid and straightforward system for transient gene expression in *E. songoricum*. This advancement enhances our ability to study *E. songoricum* specifically while providing valuable insights for the transformation of other desert woody plants.

## 2. Results

### 2.1. Development of Transient Transformation Method for E. songoricum in Seedlings

**Figure 1:**
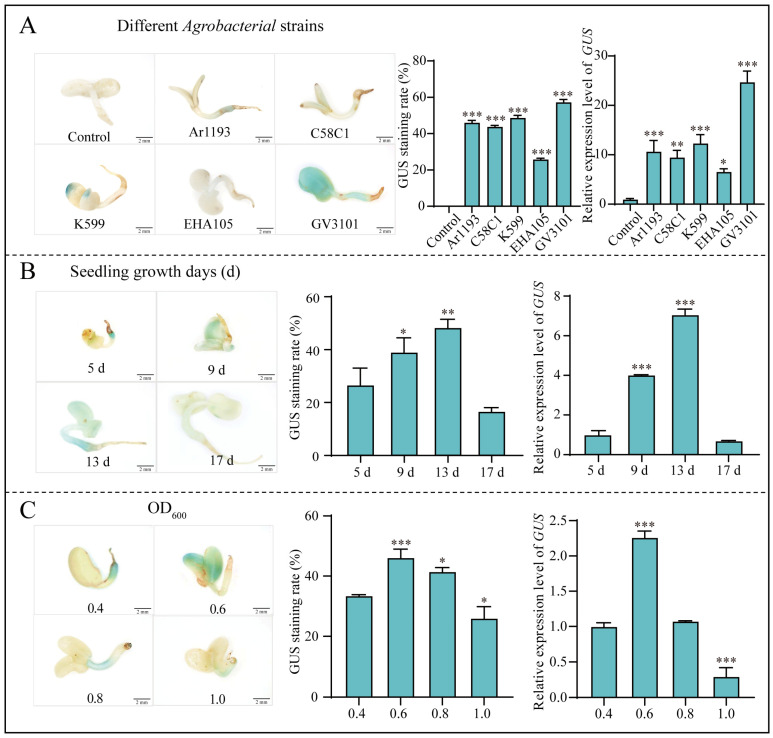
Optimization of *Agrobacterium*-mediated *E. songoricum* transformation using seedlings as explants by three evaluations of GUS staining of seedlings, relative *GUS* expression level and GUS staining rate. (**A**) Effect of different *Agrobacterium* strains (EHA105, C58C1, GV3101, Ar1193 and K599); (**B**) effect of different seedling ages (5, 9, 13 and 17 days); (**C**) effect of different *Agrobacterium* densities (OD_600_ = 0.4, 0.6, 0.8 and 1.0) on transient genetic transformation system. There were significant differences compared with the control (control, 5-day-old seedlings and OD_600_ = 0.4). Data are presented as means ± SD (3 biological replicates; *n* = 10). Asterisks indicate significant differences from the control based on Student’s *t*-test (* *p* < 0.05, ** *p* < 0.01 and *** *p* < 0.001).

In this study, we selected various *Agrobacterium* strains and seedlings at different growth stages, along with varying densities of the *Agrobacterium* transformation solution, AS concentrations, sucrose concentrations and infection times, to determine the optimal transformation conditions. The results show that *Agrobacterium* strain GV3101 was the most effective, yielding the highest expression levels of *GUS* and the highest GUS staining rate of 57.17%. The GUS staining rates of *E. songoricum* seedlings infested with *Agrobacterium* strains EHA105, C58C1, Ar1193 and K599 were 25.73%, 43.72%, 45.95% and 48.60% (Figure 1A), respectively. This indicated that GV3101 successfully introduced the *GUS* gene into the *E. songoricum* seedlings more efficiently than the other tested strains (Figure 1A). For transient genetic transformation in *E. songoricum* seedlings at 5, 9, 13 and 17 days of age (Figure 1B), the results indicate that 13-day-old seedlings had the highest *GUS* relative expression levels, with a 48.5% GUS staining rate, while the GUS staining rate at 5, 9 and 17 days were 26.6%, 39.1% and 16.6%, respectively. The *GUS* transient expression increased and then decreased with the number of days of seedling growth. Therefore, we used 13-day-old seedlings for subsequent genetic transformation experiments. Additionally, the effect of the *Agrobacterium* density on the transformation efficiency was investigated. The *GUS* expression level was the highest at an OD_600_ of 0.6 among *Agrobacterium* densities of OD_600_ = 0.4, 0.6, 0.8 and 1.0. The highest staining rate was 46.17%, which further declined with further increases in the density (Figure 1C).

**Figure 2:**
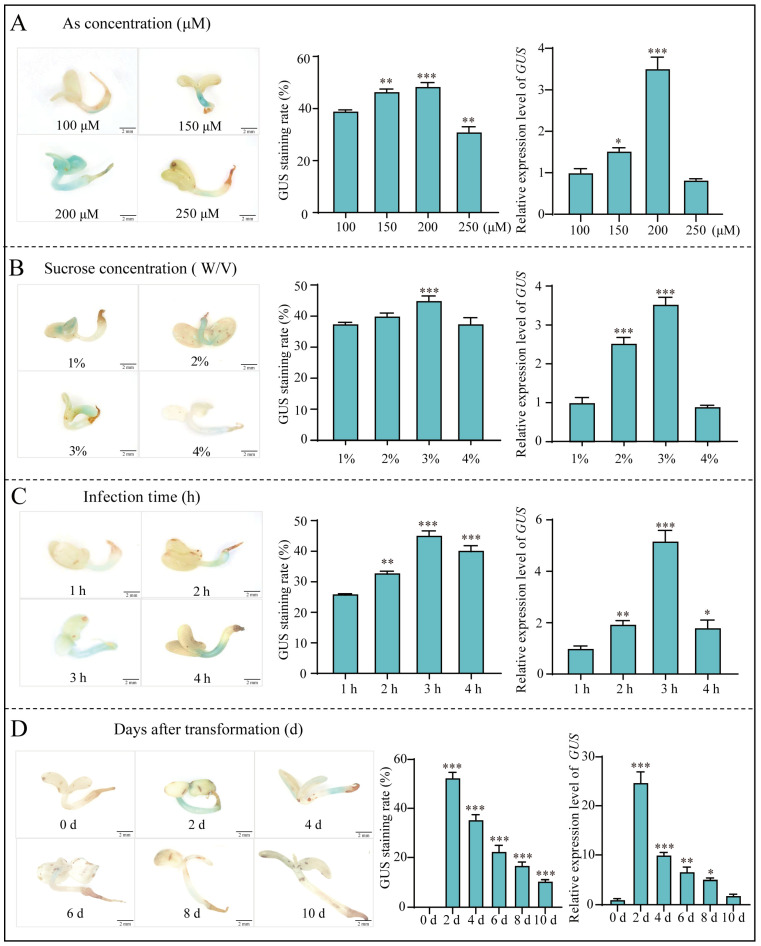
Optimization of *Agrobacterium*-mediated *E. songoricum* transformation using seedlings as explants by three evaluation of GUS staining of seedlings, relative *GUS* expression level and GUS staining rate. (**A**) effect of different AS concentrations (100, 150, 200 and 250 μM); (**B**) effect of different sucrose concentrations (1%, 2%, 3% and 4%); (**C**) effect of different infection time (1, 2, 3 and 4 h) on transient genetic transformation system; (**D**) the duration of sustained expression of *GUS* genes after transient transformation of *E. songoricum* seedlings (0, 2, 4, 6, 8 and 10 days). There were significant differences compared with the control (100 μM AS, 1% sucrose, 1 h and 0 d). Data are presented as means ± SD (3 biological replicates; *n* = 10). Asterisks indicate significant differences from the control based on Student’s *t*-test (* *p* < 0.05, ** *p* < 0.01 and *** *p* < 0.001).

Adding an appropriate amount of AS to an *Agrobacterium* resuspension can effectively improve the transformation efficiency of *Agrobacterium* [20]. In our results, when AS was added to the infection solution at concentrations of 100, 150, 200 and 250 μM, individually, the optimal staining effect was achieved at an AS concentration of 200 μM and most plants exhibited blue staining. The *GUS* transient expression rate was also the highest, with a GUS staining rate of 48.5% (Figure 2A). The sucrose concentration also affects the efficiency of transient genetic transformation [21,22]. In this study, four different sucrose concentrations (1%, 2%, 3% and 4%) of the infection solution were designed. The GUS staining results indicate that the optimal staining effect was achieved with a 3% sucrose concentration in *E. songoricum* seedlings. The GUS staining rate reached 45%. Meanwhile, the *GUS* quantitative assay revealed that the transient expression of *GUS* in *E. songoricum* initially increased and then decreased with an increased in the sucrose concentration (Figure 2B). The efficiency of transient transformation is also affected by the infection time [23]. *Agrobacterium* infection requires a specific amount of time to ensure adequate penetration into plant tissues without causing damage. The infection time was divided into four gradients: 1 h, 2 h, 3 h and 4 h. The optimal infection time was determined to be 3 h based on the data of the relative expression of *GUS* and GUS staining and the highest GUS staining rate of 46% was observed (Figure 2C). In other words, the transient expression of *GUS* in *E. songoricum* seedlings increased with longer infection times and then decreased. We investigated the expression levels of the *GUS* gene and GUS staining levels from 0, 2, 4, 6, 8 and 10 days after transient transformation to investigate the duration of the sustained expression of exogenous genes after the transient transformation of *E. songoricum* seedlings. The results indicate that the highest relative *GUS* expression and the most significant GUS staining occurred on the 2nd day post-transformation, with a staining rate of 52.47%. As time progressed, the relative expression level of the *GUS* gene gradually decreased and the staining rate of GUS decreased correspondingly (Figure 2D).

In summary, the optimal transient genetic transformation system for *E. songoricum* seedlings was as follows: 13-day seedlings were infected with *Agrobacterium* strain GV3101, using the transformation solutions composed of 1/2 MS with an OD_600_ of 0.6, 3% sucrose, 200 μM AS and an infestation duration of 3 h. The highest GUS staining rate reached 57.17%. The peak expression of the *GUS* gene occurred on the 2nd day post-transformation.

### 2.2. Development of Transient Expression Method for Assimilated Branches in E. songoricum

A rapid regeneration system of assimilated branches of *E. songoricum* was established [9], and they exhibited strong, abundant, and vigorous clonal regeneration capacity. Therefore, assimilated branches may serve as another suitable explant for establishing a transient genetic transformation system of *E. songoricum*. We conducted various influencing factors similar to those mentioned above, including different *Agrobacterium* strains, transformation solution OD_600_ values, AS concentrations, sucrose concentrations and infection times to establish a transient genetic transformation system for *E. songoricum* assimilating branches.

**Figure 3:**
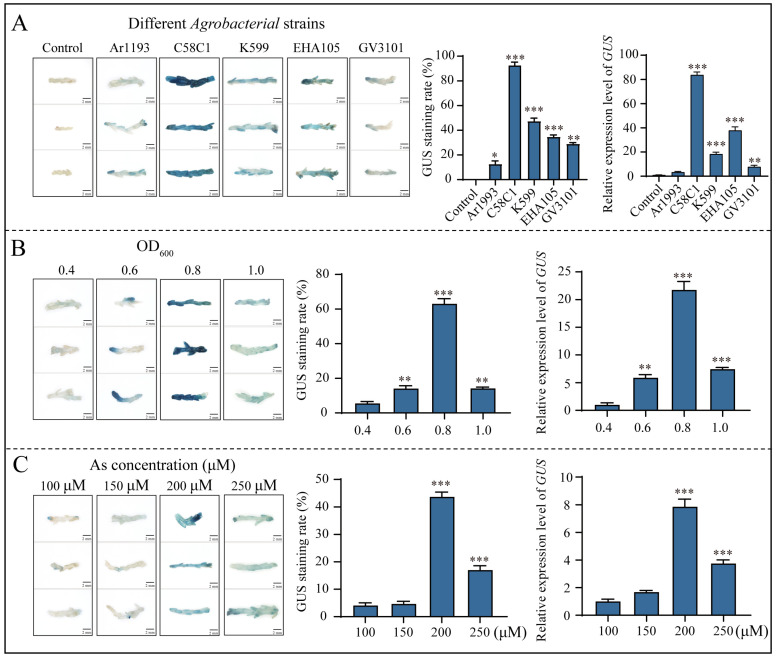
Optimization of *Agrobacterium*-mediated *E. songoricum* transformation using assimilated branches as explants using three evaluations of GUS staining of assimilated branches, relative *GUS* expression level and GUS staining rate. (**A**) Effect of different *Agrobacterium* strains (EHA105, C58C1, GV3101, Ar1193 and K599); (**B**) effect of different *Agrobacterium* density (OD_600_ = 0.4, 0.6, 0.8 and 1.0); (**C**) effect of different AS concentrations (100, 150, 200 and 250 μM.); There were significant differences compared with the control (control; OD_600_ = 0.4; 100 μM AS). Data are presented as means ± SD (3 biological replicates, *n* = 10). Asterisks indicate significant differences from the control based on Student’s *t*-test (* *p* < 0.05, ** *p* < 0.01 and *** *p* < 0.001).

The results indicate that *Agrobacterium* C58C1 was the most effective in infecting the assimilated branches, exhibiting the highest relative *GUS* expression and highest transformation efficiency of approximately 97% (Figure 3A). Therefore, we used *Agrobacterium* C58C1 for subsequent genetic transformation experiments. Furthermore, the optimal GUS staining appearance was obtained when OD_600_ = 0.8 among *Agrobacterium* densities of OD_600_ = 0.4, 0.6, 0.8 and 1.0, respectively), with a GUS staining rate of 65% and the highest relative expression of *GUS*. The lowest was at OD_600_ = 0.4, with only a 9% GUS staining rate. Almost the same GUS staining rate was achieved at OD_600_ = 0.6 and 1.0, at approximately 18% (Figure 3B). Furthermore, to confirm the AS and sucrose concentration, the infection solution was supplemented with AS at concentrations of 100, 150, 200 and 250 µM. The optimal staining effect was achieved at an AS concentration of 200 µM and the majority of the explants were stained blue, with a GUS staining rate of 45.5% and the highest relative expression level of *GUS* (Figure 3C).

**Figure 4:**
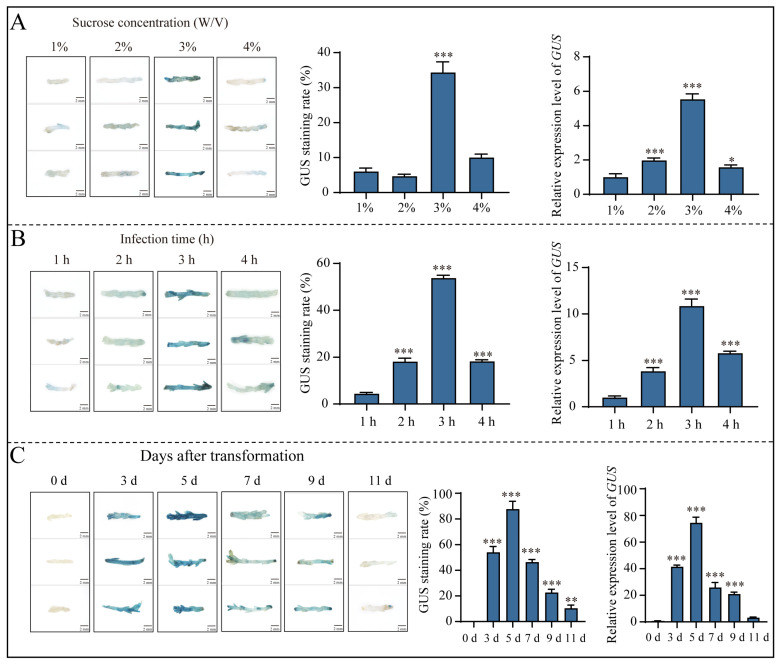
Optimization of *Agrobacterium*-mediated *E. songoricum* transformation using assimilated branches as explants using three evaluations of GUS staining of assimilated branches, relative *GUS* expression level and GUS staining rate. (**A**) Effect of different sucrose concentrations (1%, 2%, 3% and 4%); (**B**) effect of different infection time (1, 2, 3 and 4 h); (**C**) the duration of sustained expression of *GUS* genes after transient transformation of *E. songoricum* assimilated branches (0, 3, 5, 7, 9 and 11 days). There were significant differences compared with the control (1% sucrose, 1 h and 0 d). Data are presented as means ± SD (3 biological replicates; *n* = 10). Asterisks indicate significant differences from the control based on Student’s *t*-test (* *p* < 0.05, ** *p* < 0.01 and *** *p* < 0.001).

Furthermore, the infection solution was supplemented with four distinct sucrose concentrations (1%, 2%, 3% and 4%, separately). The transient expression of *GUS* in the assimilating branches initially increased with the increase in the sucrose concentration and subsequently declined. The highest transient expression of *GUS* was observed at a sucrose concentration of 3%, with a GUS staining rate of 38.5% (Figure 4A). Moreover, the highest transient expression of *GUS* and the optimal staining effect was achieved when the infection time was 3 h, with a GUS staining rate of 58% (Figure 4B). Finally, we investigated the duration of sustained expression of exogenous genes after the transient transformation of assimilating branches for 3, 5, 7, 9 and 11 days according to the levels of *GUS* gene expression and GUS staining (Figure 4C). The results show that the optimal staining effect was achieved on the fifth day after transformation, with a GUS staining rate of 92%. The expression level and staining rate of GUS decreased to the lowest on the seventh day after transformation, with a transformation rate of only 10%.

In summary, the optimal transient transformation system for *E. songoricum* assimilating branches was as follows: assimilating branches were infected with *Agrobacterium* strain C58C1 suspended in a transformation solution composed of 1/2 MS with 3% sucrose and 200 μM AS, the *Agrobacterium* density was OD_600_ = 0.8 and the infestation duration was 3 h. The highest GUS staining rate reached 97% and the highest *GUS* gene expression level was observed on the fifth day after transformation.

### 2.3. Development of Transient Expression Method for Callus in E. songoricum

**Figure 5:**
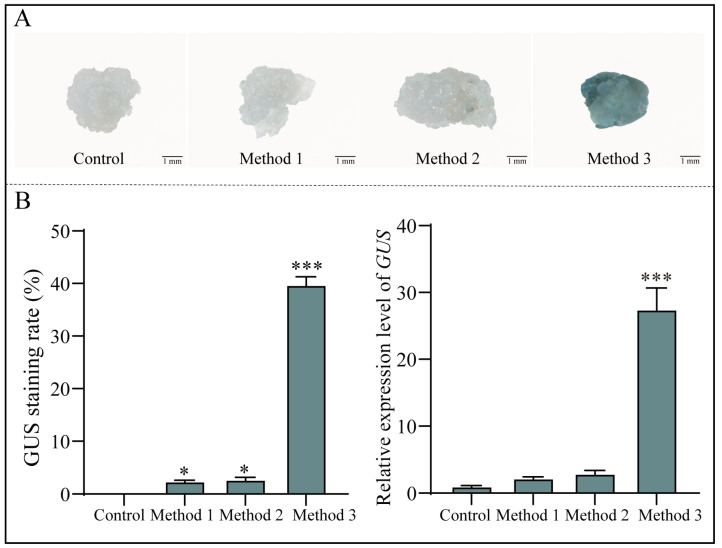
Optimization of *Agrobacterium*-mediated *E. songoricum* transformation using callus as explants. (**A**) GUS staining of three *E. songoricum* callus transient genetic transformation protocols. (**B**) Relative *GUS* expression and GUS staining rate of three *E. songoricum* callus transient genetic transformation protocols. There were significant differences compared with the control. Data are presented as means ± SD (3 biological replicates; *n* = 10). Asterisks indicate significant differences from the control based on Student’s *t*-test (* *p* < 0.05 and *** *p* < 0.001).

For the transient transformation of callus, we initially used transformation solutions similar to those used for seedlings and assimilating branches, combined with various transformation conditions (i.e., *Agrobacterium* strains, OD_600_ values, AS concentrations, sucrose concentrations and infection times). Our preliminary exploration revealed that the transformation solutions suitable for seedlings and assimilating branches were ineffective for callus. Moreover, the *Agrobacterium* strain, OD_600_ and AS concentration were also not the main factors influencing the transformation efficiency. Consequently, considering the characteristics of *E. songoricum* as a woody desert plant and the relevant literature, we explored three different approaches by adjusting the composition of the transformation solution: a conventional transformation solution (method 1), a transformation solution tailored for woody plant callus tissue (method 2) and a transformation solution designed for desert plants (method 3). We evaluated the three transformation solutions to determine the optimal transformation method, using untransformed callus as a control. The *GUS* expression and staining results demonstrated that the transformed callus did not significantly differ from the control with methods 1 and 2 (Figure 5A,B). Method 3 was employed to enhance the infection effect of *Agrobacterium*, which involved modifying the transformation solution to a 1:1 ratio of (AB: MES):MS and *Agrobacterium* underwent overnight virulence induction in AB: MES salt. This resulted in significant GUS staining with a GUS staining rate of 39.51% and a significant increase in *GUS* gene expression compared with the control (Figure 5A,B).

### 2.4. Validation of Transient Genetic Transformation System in E. songoricum

**Figure 6:**
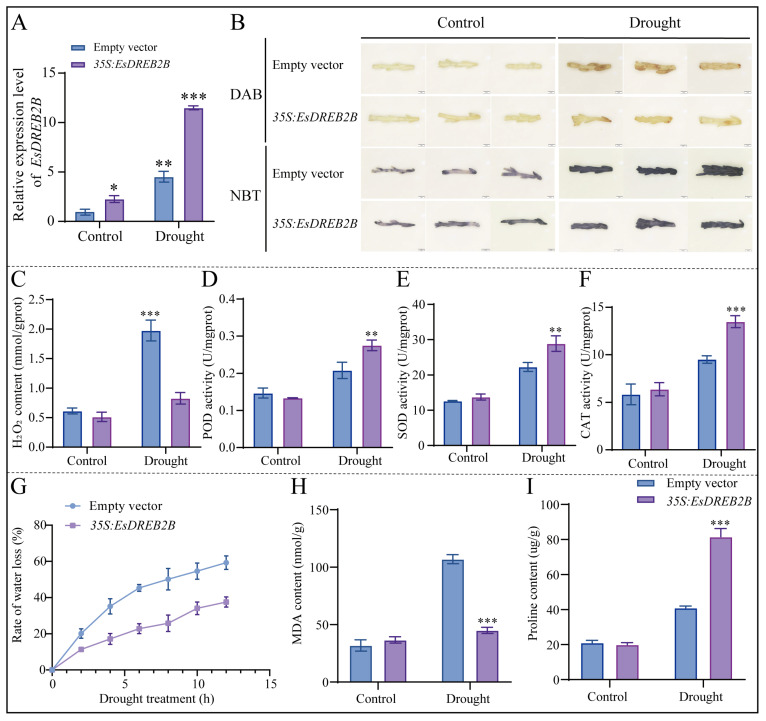
Transient expression analysis of *EsDREB2B* gene in assimilating branches of *E. songoricum* under drought treatment. (**A**) Relative expression of *EsDREB2B* gene in transiently expressed assimilating branches of *E. songoricum*; (**B**) DAB and NBT staining of assimilating branches in transformed empty vector (as control) and *EsDREB2B*-overexpressed vector under drought stress. (**C–F**) H_2_O_2_ contents, POD, SOD and CAT activities in empty vector and *EsDREB2B* overexpression assimilating branches under drought stress. (**G**) Water loss of the empty vector and *EsDREB2B* overexpression assimilating branches under drought stress; (**H**,**I**) MDA and proline contents in empty vector and *EsDREB2B* overexpression assimilating branches under drought stress. Data are presented as means ± SD (3 biological replicates; *n* = 10). Asterisks indicate significant differences from the control based on Student’s *t*-test (* *p* < 0.05, ** *p* < 0.01 and *** *p* < 0.001).

Comprehensive research showed that the highest transformation efficiency was 57.17% when using seedlings as explants, 39.51% with callus and 97% with assimilating branches in *E. songoricum*. We used assimilated branches as explant material due to their highest transformation efficiency and the *EsDREB2B* gene as an example for verification to validate the applicability of the functional verification of resistance genes using this technology for this species. The transient transformation of pCAMBIA1300-*GFP* empty vector and pCAMBIA1300-*EsDREB2B-GFP* vector into *E. songoricum* assimilating branches was performed. The qRT-PCR results indicate that *EsDREB2B* was overexpressed by 2.3-fold and upregulated by 4.5-fold and 11.5-fold, respectively, in assimilating branches of the transformed empty vector (as control) and *EsDREB2B*-overexpressed vector under drought stress (Figure 6A). Subsequently, the control transgenic assimilated branches accumulated more H_2_O_2_ and O_2_^−^, after drought stress, resulting in darker staining than the transgenic *EsDREB2B* assimilated branches. *EsDREB2B* transgenic assimilating branches significantly increased POD, SOD and CAT activities and produced less H_2_O_2_ than control assimilating branches. No significant differences were observed between the control and transgenic *EsDREB2B* assimilating branches under normal growth conditions by DAB and NBT staining, enzyme activity assays and H_2_O_2_ content determination (Figure 6B–F). Furthermore, during drought stress, the water loss rate, MDA content of *EsDREB2B* transgenic assimilating branches were significantly lower and the accumulation of osmoregulatory substances, such as proline, was notably higher than the control assimilating branches (Figure 6G–I). No significant differences were observed under normal growth conditions. These comprehensive data indicate that the overexpression of *EsDREB2B* in situ in *E. songoricum* enhanced its drought resistance, further validating the effectiveness of the transient transformation system for this species.

### 2.5. Procedure for Agrobacterium-Mediated Transient Transformation of E. songoricum

**Figure 7:**
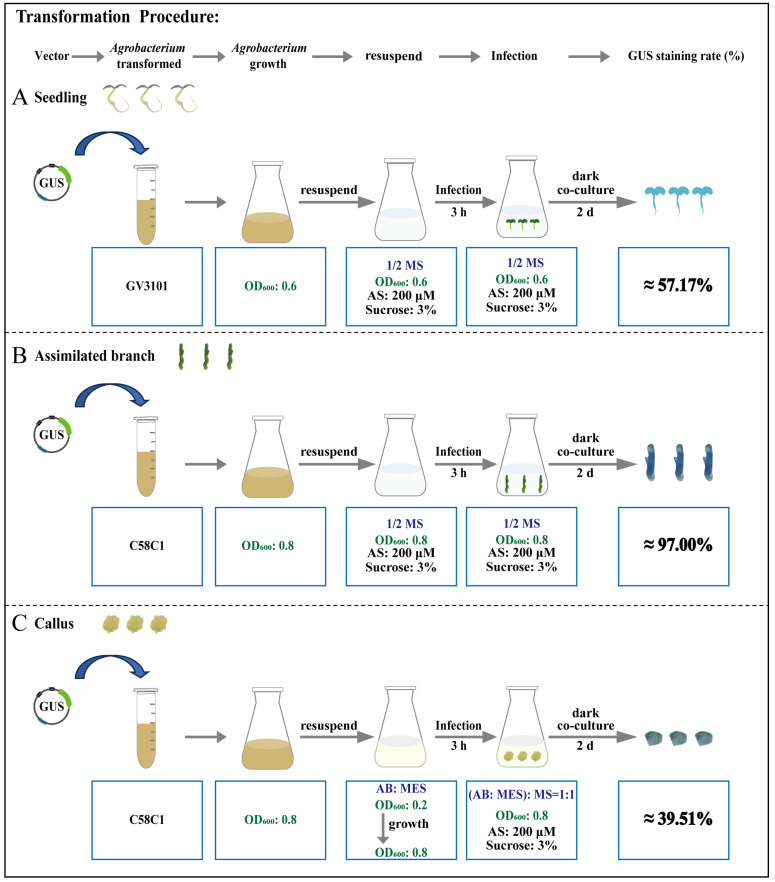
Procedure for *Agrobacterium*-mediated transient transformation of *E. songoricum*. (**A**) Procedure for *Agrobacterium*-mediated transient transformation of seedlings; (**B**) procedure for *Agrobacterium*-mediated transient transformation of assimilated branches; (**C**) procedure for *Agrobacterium*-mediated transient transformation of callus.

Our study aimed to establish an optimal transient transformation system for *E. songoricum* across three types of explants. The optimal conditions for the seedlings as explants involved infecting 13-day-old seedlings with the *Agrobacterium* strain GV3101. The infection solution consisted of 1/2 MS medium with an OD_600_ of 0.6, 3% sucrose and 200 μM AS, with an infection duration of 3 h. After two days of dark growth, transgenic seedlings were generated, reaching the peak expression of exogenous genes on the second day post-transformation. The highest transformation efficiency observed was 57.17% (Figure 7A). The best conditions for assimilating branches as explants required infecting one-month-old regenerating assimilating branches with the *Agrobacterium* strain C58C1. The infection solution comprised 1/2 MS medium with an OD_600_ of 0.8, 3% sucrose and 200 μM AS for 3 h of infection. Following two days of dark cultivation, peak gene expression appeared on the fifth day after transformation, with the maximum transformation efficiency reaching approximately 97% (Figure 7B). For the transiently transformed platform of callus: For callus derived from assimilating branches, optimal transformation was achieved by infecting 20-day-old callus with *Agrobacterium* strain C58C1. Before the transformation, *Agrobacterium* was resuspended in AB: MES medium starting at an OD_600_ of 0.2. After overnight induction, the OD_600_ reached 0.8. The infection solution was adjusted to (AB: MES):MS = 1:1 with an OD_600_ of 0.8, 3% sucrose and 200 μM AS, for a 3-h infection duration. After two days of dark growth, transgenic callus was produced, achieving a maximum transformation efficiency of 39.51% (Figure 7C). Through these experiments, we refined the conditions necessary to achieve high transient transformation efficiencies in different explants of *E. songoricum*, providing a reliable method for genetic studies in this species.

## 3. Discussion

Multiple variables influence the efficiency of *Agrobacterium*-mediated genetic transformation, including the type of explants [24,25,26], *Agrobacterium* strain [27,28], *Agrobacterium* density OD_600_ [29,30,31,32], AS concentration [33,34,35], surfactants [36] and infection time [37]. In this study, we developed an *Agrobacterium*-mediated transient transformation system using three types of explants (seedlings, assimilating branches and callus) in *E. songoricum* by exploring the effects of different *Agrobacterium* strains, seedling ages, OD_600_ values, AS concentrations, sucrose concentrations and infection times on the transformation efficiency. The most significant factors affecting the transient transformation system of *E. songoricum* were the choice of explant, the type of *Agrobacterium* strain and the composition of the transformation solution.

The choice of explant is crucial for improving the *Agrobacterium*-mediated transformation efficiency. Various explants have been used in woody plant transformation, including hairy roots [38,39,40,41], seedlings [18,42,43,44,45,46], petioles [47], stem segments [48], leaves [48], cotyledons [49,50], hypocotyls [49,51] and callus [28,52,53,54]. In our study, regarding *E. songoricum* seedlings, smaller seedlings were too tender to withstand *Agrobacterium* infection and may have suffered significant damage by post-transformation. Conversely, older seedlings had thicker, denser tissues that impeded *Agrobacterium* infection. We found that 13-day-old seedlings were optimal (Figure 1B), with cotyledons tender enough for efficient *Agrobacterium* infection, showing the highest transformation efficiency of 57.17% in *E. songoricum* (Figure 7A). Similar observations have been made in other species, such as *Populus tremula* × *P. tremuloides* [55], tomato [25] and *Jatropha curcas* [26], where younger leaves or tissues showed higher transformation efficiencies. However, *E. songoricum* is a slow-growing, endangered desert perennial woody plant, limiting the material availability for large-scale gene function verification. The assimilated branches of *E. songoricum*, as a unique tissue in woody legumes, have a strong clonal regeneration ability. Furthermore, a propagation system of assimilated branches of *E. songoricum* has been established that can rapidly obtain numerous regenerated assimilated branches within one month [9]. Consequently, the assimilated branches of *E. songoricum* can be used as a more effective explant for genetic transformation, which can be rapidly propagated, showing a maximum transient transformation efficiency of 97% (Figure 7B), 1.7 times higher than that of seedlings. This may be because the seedlings of *E. songoricum*, as a desert plant, have thicker tissues, while the assimilating branches obtained via tissue culture have younger and more tender tissues, making them more susceptible to transformation. Callus tissue had the lowest transformation efficiency of approximately 39.51% (Figure 7C), possibly due to the abundance of phenolic secondary metabolites in *E. songoricum*, which can cause tissue browning and hinder growth during transformation and callus formation [56]. Additionally, the high levels of reactive oxygen species (ROS) and phenolic compounds produced in callus may inhibit *Agrobacterium* growth, affecting its transformation efficiency [57,58]. Despite these challenges, using callus as an explant offers unique structural advantages over seedlings and assimilated branches. The development of a genetic transformation system for *E. songoricum* callus is in the initial phases of investigation. Establishing a transient genetic transformation system for callus could serve as a foundation for subsequent stable genetic transformations in *E. songoricum*.

Appropriate *Agrobacterium* strains can significantly enhance transformation efficiency [11,59]. For instance, Mahmoudian et al. compared EHA105, C58C1 and KYRT1 strains in *Leucaena leucocephala* de Wit. transformation and found KYRT1 to be 2.8 times more efficient than EHA105 and C58C1 [60]. In legume plant, EHA105 achieved a conversion efficiency of 60% in *Cicer arietinum* L. [30], K599 achieved 39% in pigeon pea [61] and Ar1193 achieved over 96% in *Glycine max* (L.) [30]. In our study, we tested five *Agrobacterium* strains (EHA105, C58C1, GV3101, Ar1193 and K599) commonly used for woody and leguminous plants. For *E. songoricum* seedlings, GV3101 showed 2.2 times higher efficiency than EHA105 and approximately 1.2 times higher efficiency than C58C1, Ar1193 and K599 (Figure 1A). For assimilating branches, C58C1 achieved the highest transformation rate of 97%, which was 5.4, 2.2, 2.6 and 2.5 times higher than those of Ar1193, K599, EHA105 and GV3101, respectively (Figure 3A). C58C1 was the most effective *Agrobacterium* strain for infesting assimilated branches of *E. songoricum*. Similar to soybean, *Agrobacterium rhizogenes* was used to achieve more than 90% transformation, indicating that *Agrobacterium rhizogenes* is more suitable for the transformation of leguminous plants.

*E. songoricum*, a woody desert plant, presents unique challenges for genetic transformation due to its callus tissue’s rich content of phenolic secondary metabolites. These compounds can lead to tissue browning, hindering growth and potentially inhibiting *Agrobacterium* growth, thereby affecting its transformation efficiency. Our research revealed that the choice of transformation solution was the primary factor influencing the callus transformation efficiency, surpassing the impact of other factors such as the *Agrobacterium* strain, OD_600_ and AS concentration. We evaluated three distinct transformation solutions based on these species-specific characteristics. Method 1 employed a conventional approach using a 1/2 MS medium-based transformation solution, which is commonly used in plant transformation [19]. While the diluted MS medium (1/2 strength) was intended to reduce stress on the callus tissue, it proved insufficient for successful transformation in *E. songoricum*. Method 2 utilized a transformation solution specifically designed for woody plant callus, comprising MS medium supplemented with plant growth regulators (TDZ and NAA) [53,54,61]. The addition of these growth regulators aimed to promote cell division and growth in the callus during the transformation process, but the transformation efficiency of this method was not ideal. Method 3 adopted a two-step approach that successfully transformed another desert plant species [59,62]. This method involved first culturing *Agrobacterium* in AB: MES medium overnight to induce virulence, followed by using a 1:1 mixture of AB: MES and MS media as the transformation solution. The AB: MES medium, rich in phosphates and nitrogen sources, provided a balanced nutrient profile that supported both *Agrobacterium* infection and callus viability while potentially mitigating the negative effects of phenolic compounds. The overnight culture allowed for the activation of virulence genes before exposure to the plant tissue, promoting *Agrobacterium*’s growth and virulence, thereby potentially increasing its transformation efficiency.

## 4. Materials and Methods

### 4.1. Plant Material

*E. songoricum* seeds were collected from the Gurbantunggut Desert in Xinjiang, China (88°24′67″ E, 45°58′11″ N) [7]. Sterilized seeds were evenly distributed on moist filter paper and sprayed with water daily to obtain seedlings. Seedlings grown for 13 days were selected as explants (Figure S1A). For assimilated branches, sterilized seeds were placed on Murashige and Skoog (MS) medium (30 g/L sucrose and 6.0 g/L agar; pH 5.8). The seeds germinated after 3 days and assimilating branches began to emerge after 11 days. Assimilated branches cultured for 40 days were cut into 1 cm long segments and placed on fresh MS medium. After 30 days of regeneration, these assimilated branches were used as explants (Figure S1B). The seeds on moist filter paper and the MS medium were cultured at 24 ± 1 °C under a 16 h light/8 h dark photoperiod with an illumination of a 100 µmol m^−2^ s^−1^ light intensity and 45% relative humidity [9]. For callus formation, 40-day-old assimilating branches were cut into 1 cm long segments and cultured on a callus induction medium for 15 days. The resulting a callus was then transferred to callus regeneration medium. The callus regenerated for 20 days and was selected for use as the explant (Figure S1C). The callus was cultured at 24 ± 1 °C under dark conditions with 50% relative humidity [9].

### 4.2. Vector Construction and Transformation by Agrobacterium

The plant expression vector pCAMBIA1301-*GUS* was transformed into the *Agrobacterium tumefaciens* EHA105 and GV3101 and *Agrobacterium rhizogenes* C58C1, Ar1193 and K599 strains. The full-length CDS of *EsDREB2B* without a stop codon was cloned and inserted into the 5′ end of the *GFP* sequence driven by the CaMV35S promoter in vector pCAMBIA1300-*GFP* to produce the fusion expression vectors pCAMBIA1300-*EsDREB2B-GFP*. pCAMBIA1300-*GFP* and the fusion expression vectors pCAMBIA1300-*EsDREB2B-GFP* were transformed into the *Agrobacterium rhizogenes* C58C1 strain. The pCAMBIA1300-35S-*GFP* vector was used as the control. The primers used for the CDS full-length clone and vector construction of *EsDREB2B* are shown in Table S1.

### 4.3. Transient Transformation Procedure for E. songoricum Seedlings and Assimilated Branches

Various factors can influence *Agrobacterium*-mediated transformation efficiency, including the plant’s physiological condition, different *Agrobacterium* strains, the *Agrobacterium* density, the infection time and AS and sucrose concentrations [11,27,28,59]. For seedlings as explants, we selected various *Agrobacterium* strains (EHA105, GV3101, C58C1, K599 and Ar1193) and seedlings at different growth stages (5, 9, 13 and 17 days), along with varying densities of the *Agrobacterium* transformation solution (OD_600_ = 0.4, 0.6, 0.8 and 1.0), AS concentrations (100 μM, 150 μM, 200 μM and 250 μM), sucrose concentrations (1%, 2%, 3% and 4%; *w*/*v*) and infection times (1 h, 2 h, 3 h and 4 h) to determine the optimal transformation conditions. Similarly, for assimilating branches, we used similar transformation conditions as for the seedlings, including the selection of *Agrobacterium* strains, transformation solution OD_600_ values, AS and sucrose concentrations and infection times to identify the best transformation efficiency. We conducted experiments focusing on these factors using the pCAMBIA1301-*GUS* vector [19]. *Agrobacterium* strains with vectors pCAMBIA1301-*GUS* were used for the transient transformation of *E. songoricum* seedlings and assimilated branches [19]. The procedure was as follows: *Agrobacterium* liquid cultures were grown at 28 °C overnight in LB medium until the OD_600_ reached 0.4–0.6. *Agrobacterium* was collected via centrifugation and re-suspended into transformation solutions. The transformation solutions comprised 1/2 MS medium with AS, sucrose, 10 mM MES and 10 mM MgCl_2_. The seedlings and assimilating branches were placed in the transformation solutions, where they were infected for 3 h (90 rpm; 25 °C) [19]. Then, transgenic seedlings or assimilated branches were generated after 2 days of growth in the dark.

### 4.4. Transient Transformation Procedure for E. songoricum Callus

Based on literature reports and exploratory research, we selected *Agrobacterium rhizogenes* strain C58C1 containing the vector pCAMBIA1301-*GUS* for callus transformation and three transformation solutions were used to evaluate the optimal transformation system.

Method 1: The transformation solution comprised 1/2 MS medium with OD_600_ = 0.8, 200 μM AS, 3% sucrose, 10 mM MES and 10 mM MgCl_2_ [19].

Method 2: The transformation solution comprised MS medium with OD_600_ = 0.8, 200 μM AS, 3% sucrose, 2.0 mg/L thidiazuron (TDZ), 0.5 mg/L α-naphthalene acetic acid (NAA), 10 mM MES and 10 mM MgCl_2_ [61,63].

Method 3: Before the transformation solution was prepared, *Agrobacterium* was resuspended in AB: MES salts (17.2 mM K_2_HPO_4_, 8.3 mM NaH_2_PO_4_, 18.7 mM NH_4_Cl, 2 mM KCl, 1.25 mM MgSO_4_, 100 μM CaCl_2_, 10 μM FeSO_4_, 50 mM MES and 2% glucose [*w*/*v*]; pH 5.5) with an initial OD_600_ of 0.2. The culture was incubated overnight at 28 °C until it reached an OD_600_ of 0.8. The next day, the *Agrobacterium* was resuspended in a transformation solution consisting of (AB: MES):MS = 1:1 with OD_600_ = 0.8, 200 μM AS and 3% sucrose [59,62].

*E. songoricum* callus samples were then placed in each of the three transformation solutions and infected for 3 h (90 rpm; 28 °C). The callus was washed three times with sterile ddH_2_O for two minutes each time. The sterile callus was then transferred onto a co-culture medium containing MS medium, 2.0 mg/L TDZ, 0.5 mg/L NAA, 6 g/L agar and 200 μM AS. After 2 days of co-culture under dark conditions, the transgenic callus was generated.

### 4.5. Histochemical Detection of GUS Activity

According to Jefferson et al. ‘s method [23], we performed GUS activity histochemical staining on the tissue samples. The explants of *E. songoricum* (seedlings, assimilated branches and callus) were immersed in GUS staining solution containing the following components: 19.5 mM NaH_2_PO_4_·H_2_O, 30.5 mM Na_2_HPO_4_·H_2_O (pH 7.0), 50 mM K_3_Fe (CN)_6_, 50 mM K_4_Fe (CN)_6_, 100 mM Na_2_EDTA (pH 7.0), 0.1% Triton X-100 and 0.6 mg/mL X-gluc. The explants were then incubated at 37 °C for 6–12 h to allow for staining. After staining, the explants were decolorized using anhydrous ethanol. The stained explants were observed and photographed under a stereomicroscope. Explants that stained blue were considered GUS-positive, indicating successful transformation. Wild-type plants were used as controls for comparison.

### 4.6. Physiological and Enzymatic Analysis of Transgenic Assimilated Branches Under Natural Drought

The empty vector and *EsDREB2B*-overexpressing assimilated branches were subjected to natural drought via air-flow on the bench in lab conditions (relative humidity: 30–40%; 24 ± 1 °C) for 12 h. Starting at 0 h, the samples were weighed every 2 h to measure the water loss. Additionally, samples were collected at both 0 h (control) and 12 h (drought) for physiological and enzymatic analyses. Histochemical staining with DAB and NBT was employed to analyze the accumulation of H_2_O_2_ and O_2_^−^ anions, following Xi et al.’s methodology [64]. Physiological indexes including H_2_O_2_, POD, SOD and CAT activities were measured using detection assay kits (kit no. A064-1, A084-3, A001-1 and A007-1; Nanjing Jiancheng Bioengineering Institute, Nanjing, China) based on the manufacturer’s instructions. The malondialdehyde (MDA) and proline contents were determined using the Nanjing Jiancheng kit (kit no. A003-4, A064-1; Nanjing Jiancheng Bioengineering Institute, China) following the manufacturer’s instructions.

### 4.7. RNA Isolation and PCR Analysis

RNA was extracted from the seedlings, assimilated branches and callus using the E.Z.N.A.^®^ Total RNA Kit I (OMEGA, Norwalk, CT, USA) following the instructions. Then, 1 μg of total RNA was reverse-transcribed to cDNA using the PrimeScript™ RT Reagent Kit with gDNA Eraser (Perfect Real Time) (Takara, Osaka, Japan). Real-time quantitative PCR analysis (RT-qPCR) using the PrimeScript™ RT Reagent Kit with gDNA Eraser (Perfect Real Time) (Takara, Japan) was run in Bio-Rad CFX96 (Applied Biosystems, Waltham, MA, USA). The reaction details were: 95 °C for 30 s, followed by 39 cycles at 95 °C for 5 s, 60 °C for 30 s and 72 °C for 20 s. Raw RT-qPCR date was acquired using QuantStudio™ Design & Analysis SC Software v1.0. *EsActin* was used as an internal control [65]. The relative expression levels of the candidate genes were calculated with the formula 2^−∆∆Ct^. [66] The primers are listed in Table S2. All reactions were performed with three biological replicates.

### 4.8. Statistical Analysis

The data were analyzed using one ANOVA and Student’s *t*-test using the GraphPad Prism 7 software. The data were presented as the means ± SD of the replicates. The asterisks indicated significant differences (* represents *p* < 0.05, ** represents *p* < 0.01 and *** represents *p* < 0.001).

## 5. Conclusions

We reported an *Agrobacterium*-mediated transient transformation system using the *GUS* gene as a marker. We investigated three types of explants (seedlings, assimilated branches and callus) and explored the effects of different *Agrobacterium* strains, seedling ages, OD_600_ values, AS and sucrose concentrations and infection times on the transformation efficiency. The results revealed that the highest transformation efficiency achieved was 57.17% for seedlings, 97% for assimilated branches and 39.51% for callus. We successfully conducted an in situ functional validation of *EsDREB2B*’s role in drought resistance utilizing the best efficient transient transformation system developed for assimilated branches. This accomplishment underscores the system’s effectiveness in studying gene functions in *E. songoricum*. This system will aid in exploring the genetic resources related to stress resistance in *E. songoricum*. Moreover, it provides valuable insights and serves as a reference for the transformation of other desert and woody plant species.

## Data Availability

Enquiries regarding the original contributions presented in the study can be directed to the corresponding author.

## Supplementary Materials

The following supporting information can be downloaded at: https://www.mdpi.com/article/10.3390/ijms252211934/s1.
