# Supplementary material for: Establishment of Agrobacterium-Mediated Transient Transformation System in Desert Legume Eremosparton songoricum (Litv.) Vass
Source: Int J Mol Sci. 2024 Nov 6;25(22):11934. doi: 10.3390/ijms252211934 (PMC11593363; doi:10.3390/ijms252211934)
Supplement: Supplementary file 1 [file ijms-25-11934-s001.zip › ijms-3285162-supplementary.pdf]

Table S1 The primers used for vector construction

Gene Name	Primer names	length	Sequence (5′→3′)
pCAMBIA1300	pCAMBIA1300-Xba	621bp	TGAGGATCCGAGCTCTCTAGAATG
-*EsDREB2B*	I-EsDREB2B-F		AGTGCAACTTGCATGCACA
pCAMBIA1300	pCAMBIA1300-Xba		CAGGTCGACGAATTCTCTAGAAGA
-*EsDREB2B*	l-EsDREB2B-R		CAATGAAGGATCCCAGCAA

Table S2 The primers used for Real-time quantitative PCR

Gene Name	Primer names	length	Sequence (5′→3′)
GUS	GUS-F	116bp	CTGGCAACCGGGTGAAGGTT
GUS	GUS-R		GTTGGCCCTTCACTGCCACT
EsDREB2B	EsDREB2B-F	222bp	CCGAGAATTCGCGCTGTAAC
EsDERB2B	EsDREB2B-R		AAGCTTTTGAAGCTTCCAGCTG
EsActin	EsActin-F	503bp	AGACCACCAAGTACTACTGCAC
EsActin	EsActin-R		CCACCAATCTTGTACACATCC

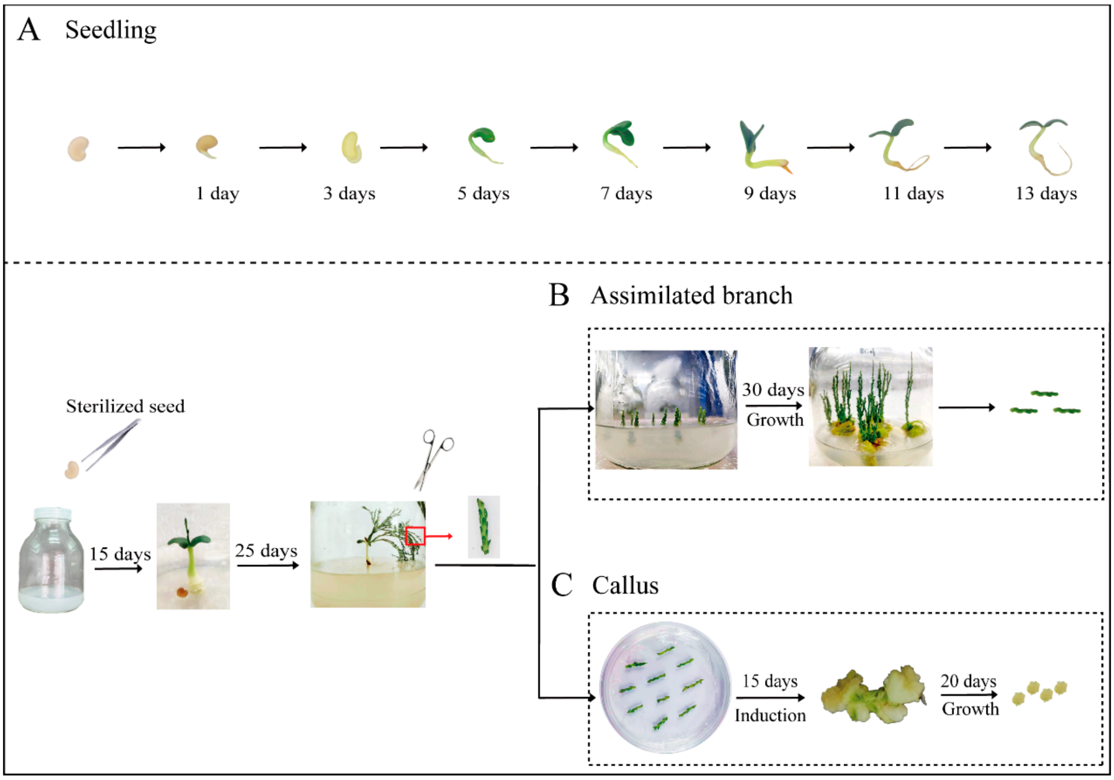

Figure S1. Methods of obtaining seedlings, assimilated branches and callus explants in *E. songoricum*. (**A**) Growth of *E. songoricum* from seeds to 13-day-old seedlings. (**B**) Obtaining assimilated branches of *E. songoricum*. (**C**) Obtaining *E. songoricum* callus.
